# Mapping of mitral regurgitant defects by cardiovascular magnetic resonance in moderate or severe mitral regurgitation secondary to mitral valve prolapse

**DOI:** 10.1186/1532-429X-10-16

**Published:** 2008-04-09

**Authors:** Ruvin S Gabriel, Andrew J Kerr, Owen C Raffel, Ralph A Stewart, Brett R Cowan, Christopher J Occleshaw

**Affiliations:** 1 Department of Cardiology, Middlemore Hospital, Auckland, New Zealand; 2 GreenLane Cardiovascular Service, Auckland City Hospital, Auckland, New Zealand; 3 Centre for Advanced Magnetic Resonance Imaging, University of Auckland, Auckland, New Zealand

## Abstract

**Purpose:**

In mitral valve prolapse, determining whether the valve is suitable for surgical repair depends on the location and mechanism of regurgitation. We assessed whether cardiovascular magnetic resonance (CMR) could accurately identify prolapsing or flail mitral valve leaflets and regurgitant jet direction in patients with known moderate or severe mitral regurgitation.

**Methods:**

CMR of the mitral valve was compared with trans-thoracic echocardiography (TTE) in 27 patients with chronic moderate to severe mitral regurgitation due to mitral valve prolapse. Contiguous long-axis high temporal resolution CMR cines perpendicular to the valve commissures were obtained across the mitral valve from the medial to lateral annulus. This technique allowed systematic valve inspection and mapping of leaflet prolapse using a 6 segment model. CMR mapping was compared with trans-oesophageal echocardiography (TOE) or surgical inspection in 10 patients.

**Results:**

CMR and TTE agreed on the presence/absence of leaflet abnormality in 53 of 54 (98%) leaflets. Prolapse or flail was seen in 36 of 54 mitral valve leaflets examined on TTE. CMR and TTE agreed on the discrimination of prolapse from flail in 33 of 36 (92%) leaflets and on the predominant regurgitant jet direction in 26 of the 27 (96%) patients. In the 10 patients with TOE or surgical operative findings available, CMR correctly classified presence/absence of segmental abnormality in 49 of 60 (82%) leaflet segments.

**Conclusion:**

Systematic mitral valve assessment using a simple protocol is feasible and could easily be incorporated into CMR studies in patients with mitral regurgitation due to mitral valve prolapse.

## Introduction

Mitral valve repair is preferred over prosthetic valve replacement for patients with mitral valve prolapse and symptomatic severe mitral regurgitation as it avoids the need for chronic anti-coagulation, has lower surgical mortality and results in better survival and long-term left ventricular function [1,2]. Mitral valve repair but not valve replacement, may also be reasonable for asymptomatic patients with severe mitral regurgitation and preserved left ventricular function if the probability of successful repair is greater than 90% [3]. Deciding if successful mitral valve repair is likely depends on accurately determining the location of leaflet abnormality, mechanism of regurgitation and the presence of a flail leaflet. Currently, 2-dimensional TTE is the first line investigation for assessing the mechanism of mitral regurgitation and identifying which leaflet is involved. However, when uncertainty is present, systematic mapping of the leaflets with TOE may be required [4,5].

CMR using cine images with optimised spatial and temporal resolution can also resolve mitral valve leaflet structure and motion, and can identify regurgitant jet direction. However, systematic mapping of mitral regurgitation in patients with mitral valve prolapse using CMR has not been described. In this study, we assess the feasibility of a simple CMR mapping protocol to provide an accurate anatomic map of leaflet abnormalities and identify mitral regurgitant jet direction.

## Methods

### Study population and protocol

Patients with isolated moderate to severe mitral regurgitation due to myxomatous mitral valve prolapse and normal left ventricular function were invited to participate. All patients were prospectively recruited from cardiology outpatient clinics and enrolled between June 2005 and December 2006. Exclusion criteria included the presence of other significant valvular disease, atrial fibrillation or flutter, prior coronary artery disease, poor echocardiographic images or inability to undergo a CMR scan. Each patient underwent CMR followed by TTE on the same day. TOE or surgery was performed within 6 months in 10 patients. The study protocol was approved by the regional ethics committee and all subjects gave written informed consent.

### Cardiac magnetic resonance image acquisition

**Figure 1:**
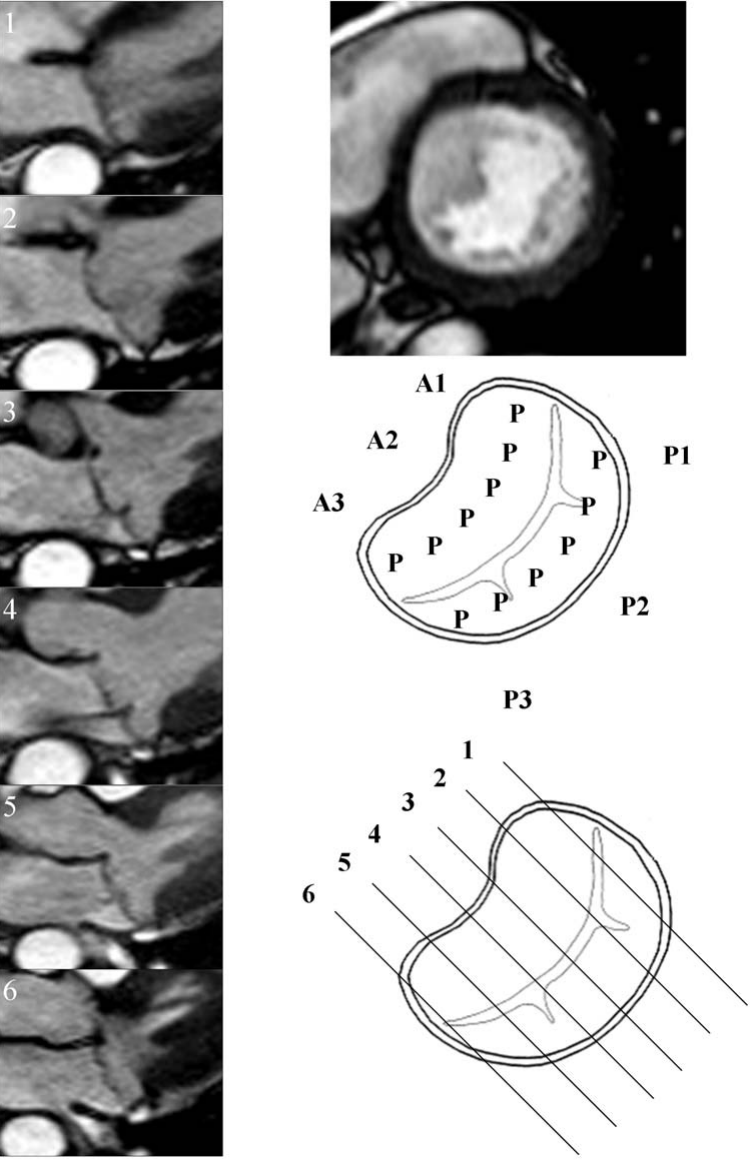
**Bi-leaflet mitral valve prolapse.** Mitral valve mapping by CMR. N – Normal leaflet segment, P – Prolapsing leaflet segment, F-Flail leaflet segment.

**Figure 2:**
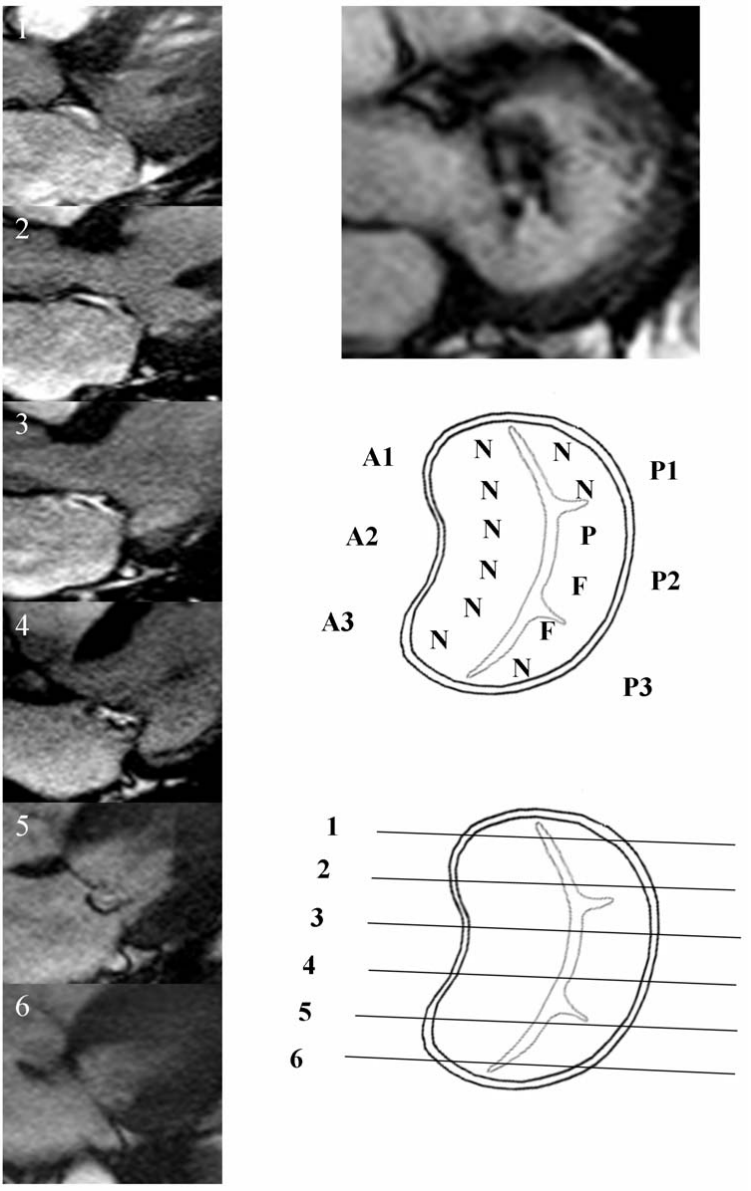
**Posterior mitral valve prolapse.** Mitral valve mapping by CMR. N – Normal leaflet segment, P – Prolapsing leaflet segment, F-Flail leaflet segment.

**Figure 3:**
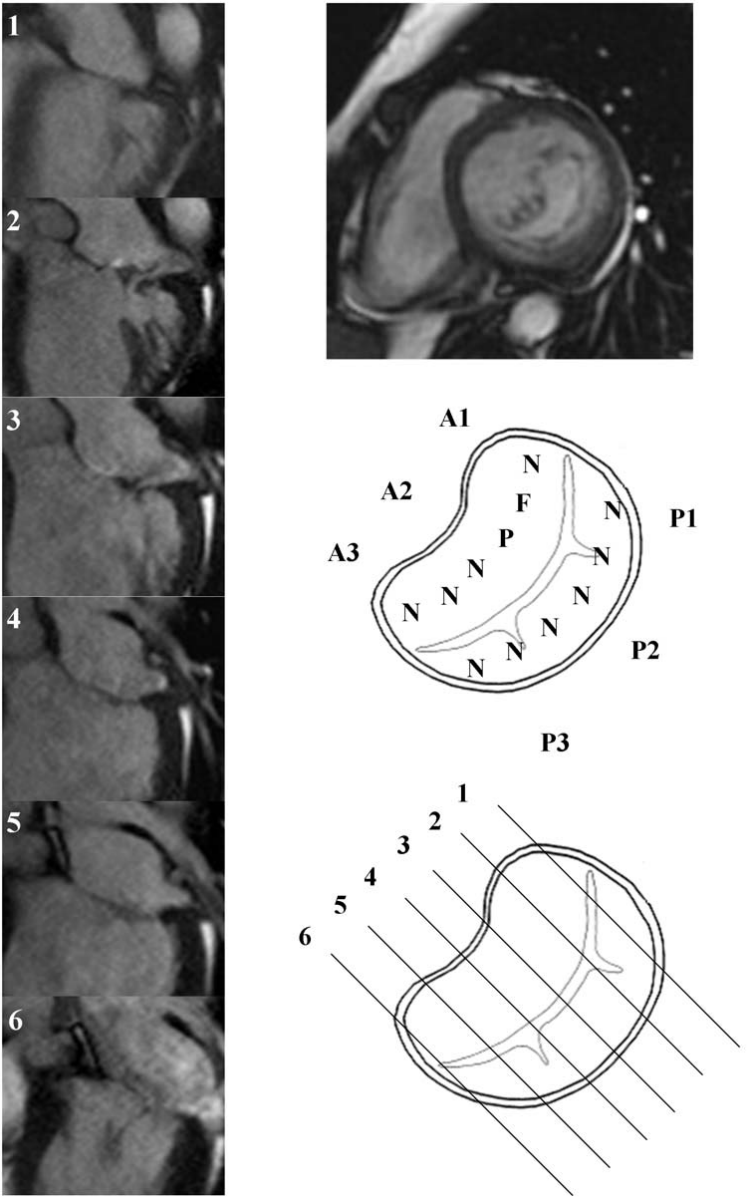
**Anterior mitral valve prolapse.** Mitral valve mapping by CMR. N – Normal leaflet segment, P – Prolapsing leaflet segment, F-Flail leaflet segment.

All patients underwent CMR on a Siemens Avanto 1.5 Tesla machine (Siemens Medical Systems, Erlangen, Germany) with a phased array body coil. A basal short axis slice through the mitral valve leaflets was planned from long axis steady state free precession (true FISP) scout cine images. Contiguous (slice thickness 6 mm) long-axis cine images perpendicular to the valve commissures were obtained across the mitral valve from the medial to lateral annulus (Figures 1, 2 and 3). High temporal resolution cine imaging was optimised by maximising the number of phases per R-R interval for a given TR. Spatial resolution was optimised by minimising the field of view. Typical imaging parameters were TR/TE 2.90/1.22 msec, 62° flip angle, 256 × 208 image matrix, field of view 390 × 317 mm, slice thickness 6 mm, 8 segments, iPAT factor 2, bandwidth 930 Hz/pixel, one slice per breath-hold with temporal resolution of 23 msec.

Systematic valve inspection and mapping using a 6 segment model was undertaken[5]. The Carpentier nomenclature was applied to the mitral valve leaflets [6]. Each leaflet was divided into three sections [5]. The anterior leaflet was defined as: A1 – lateral segment, A2 – middle segment and A3 – medial segment. The posterior leaflet was defined as: P1 – lateral segment, P2 – middle segment and P3 – medial segment. For a typical patient 6 to 7 contiguous long axis slices were obtained perpendicular to the valve commissures. For analysis the two most lateral slices were defined as the 1-level segments, the two most medial slices were defined as the 3-level segments and the remaining central slices were considered to be 2-level segments. Appropriate allocation of slices to segments was checked by visualising slice position on the basal short axis view of the valve. Each leaflet segment was classified as normal, prolapse or flail (Figure 1). Valve prolapse was defined as extension of the mitral valve leaflet ≥ 2 mm beyond the mitral annular plane in the 3 chamber long axis view [7]. A flail leaflet segment was defined as any extension of the mitral valve leaflet tip horizontal to or away from the atrial aspect of the mitral annular plane [7]. The number of flail segments was quantified. Based on the segmental findings, each leaflet was defined as normal or abnormal and the mitral valve prolapse classified as anterior, posterior or bi-leaflet prolapse. The regurgitant jet direction was determined from the jet signal flow void seen on the long axis cine images.

### Trans-thoracic echocardiography assessment

Each patient underwent a TTE by an experienced sonographer according to a standardised protocol using a conventional echo machine (Phillips HDi5000 or Phillips IE33, Andover, MA, USA). The mitral valve apparatus was carefully assessed in 4 standard views (para-sternal long axis, apical-4 chamber, apical-3 chamber and apical-2 chamber views), with and without colour Doppler imaging of the mitral regurgitant jet. On TTE each leaflet was not divided into individual segments, but was classified as normal, prolapse or flail present. As with the CMR analysis, each mitral valve prolapse was classified as anterior, posterior or bi-leaflet prolapse. The regurgitant jet direction was assessed from colour Doppler imaging from the 4 standard views. Mitral regurgitation severity was classified according to standard criteria[8].

### Trans-oesophageal echocardiography and surgical findings

7 patients, enrolled in the study, had a TOE performed by experienced cardiologists as part of their clinical evaluation. The results were compared with the TTE and CMR findings. 3 patients proceeded to mitral valve surgery during the study enrolment period. Operative findings were compared with the TTE and CMR findings in these patients.

### Inter-observer and intra-observer variability

10 mitral valve studies were independently evaluated by 2 cardiologists for inter-observer variability and re-evaluated by one cardiologist for intra-observer variability.

## Results

### Study population

27 patients with mitral regurgitation due to valve prolapse had paired TTE and CMR scans. Suitable image quality was obtained in all subjects. The mean age of the study population was 60.7 years. Mitral regurgitation was of moderate severity in 11 patients and severe in 18 patients. There was isolated anterior mitral valve leaflet prolapse in 2 patients, isolated posterior leaflet prolapse in 16 patients and bi-leaflet prolapse in 9 patients by TTE. 36 of 54 leaflets had prolapsing or flail segments (Table 1). On average 6.9 long-axis slices through each mitral valve were obtained with CMR imaging.

**Table 1:** Classification of mitral valve prolapse by cardiac magnetic resonance imaging and trans-thoracic echocardiography

	**PMVL**	**AMVL**	**CMR Classification**	**PMVL**	**AMVL**	**TTE Classification**	**Disagree**	**Reason**
Patient 1	2	1	Bi-leaflet	2	1	Bi-leaflet		
Patient 2	2	0	Posterior	2	0	Posterior		
Patient 3	1	1	Bi-leaflet	1	1	Bi-leaflet		
Patient 4	2	0	Posterior	2	0	Posterior		
Patient 5	1	2	Bi-leaflet	1	2	Bi-leaflet		
Patient 6	0	1	Anterior	0	2	Anterior	Disagree	CMR = AMVL prolapse, TTE = AMVL flail
Patient 7	2	0	Posterior	2	0	Posterior		
Patient 8	1	0	Posterior	1	0	Posterior		
Patient 9	2	0	Posterior	2	0	Posterior		
Patient 10	1	0	Posterior	1	0	Posterior		
Patient 11	1	0	Posterior	1	0	Posterior		
Patient 12	0	2	Anterior	0	1	Anterior	Disagree	CMR = AMVL flail, TTE = AMVL prolapse
Patient 13	1	0	Posterior	1	0	Posterior		
Patient 14	2	0	Posterior	2	0	Posterior		
Patient 15	2	0	Posterior	2	0	Posterior		
Patient 16	1	1	Bi-leaflet	2	1	Bi-leaflet	Disagree	CMR = PMVL prolapse, TTE = PMVL flail
Patient 17	1	0	Posterior	1	0	Posterior		
Patient 18	1	1	Bi-leaflet	1	1	Bi-leaflet		
Patient 19	1	1	Bi-leaflet	1	1	Bi-leaflet		
Patient 20	1	1	Bi-leaflet	1	1	Bi-leaflet		
Patient 21	1	0	Posterior	1	0	Posterior		
Patient 22	1	1	Bi-leaflet	1	1	Bi-leaflet		
Patient 23	1	1	Bi-leaflet	1	1	Bi-leaflet		
Patient 24	1	0	Posterior	1	0	Posterior		
Patient 25	2	0	Posterior	2	0	Posterior		
Patient 26	1	0	Posterior	1	0	Posterior		
Patient 27	2	0	Posterior	2	1	Bi-leaflet	Disagree	CMR = normal AMVL, TTE = AMVL prolapse

0 – Normal leaflet, 1 – Prolapsing leaflet, 2 – Flail leaflet

### Leaflet abnormalities and jet direction; comparison of CMR and TTE findings

CMR and TTE agreed on the presence/absence of leaflet abnormality in 53 of 54 (98%) leaflets. Of these 53 leaflets the CMR and TTE disagreed regarding whether the abnormality was prolapse or flail for 3 leaflets. CMR and TTE agreed on the discrimination of prolapse from flail in 33 of 36 (92%) leaflets. Of 12 flail leaflets seen on TTE, 10 were visualised on CMR. Of the 2 flail leaflets not visualised by CMR, one was classified as prolapse on surgical inspection. CMR detected one eccentric flail segment not detected by TTE. CMR and TTE agreed on the predominant regurgitant jet direction in 26 of the 27 patients.

### Segmental mapping of leaflet abnormalities; comparison with TOE and surgical findings

In comparison with 10 patients with TOE or surgical operative findings, CMR mapping of the mitral valve correctly classified 49 of 60 segments (82%) (Table 2) according to whether there was a leaflet abnormality involving a particular segment. There were three segments in which both techniques identified an abnormality but in which there was disagreement regarding whether the abnormality was prolapse or flail. On re-examination of the 11 non-concordant segments the disagreements were predominantly regarding minor degrees of prolapse (2 to 3 mm). TOE identified mild prolapse of the AMVL leaflet let tip in three cases not seen by CMR, and CMR identified mild prolapse of the lateral (1 level) or medial (3 level) in seven patients not seen by TOE/surgery. One patient had A2 seen at surgery only, so could not be reassessed.

**Table 2:** Classification of mitral valve prolapse by cardiac magnetic resonance imaging versus trans-esophageal echocardiography and/or operative findings

	**A3**	**A2**	**A1**	**P3**	**P2**	**P1**	**TEE/Surgery**	**Segment agreement**
Patient 1	0	1	0	1	2	1	Surgery: P2 flail and A2 prolapse	6
Patient 4	0	0	0	0	2	1	TEE: P2 prolapse with P3 prolapse, flail cord	5
Patient 5	1	2	0	1	1	1	TEE: A2 flail, PMVL prolapsed, Surgery: A2 flail, PMVL prolapse	6
Patient 7	0	0	0	0	2	1	TEE: P2 flail cord rupture, mild P1 prolapse	6
Patient 8	0	0	0	1	1	0	TEE: P2 prolapse, mild mid anterior prolapse	5
Patient 9	0	0	0	0	2	1	Surgery: P2 flail, P1 flail	6
Patient 14	0	0	0	2	1	0	TEE: Post flail P2, P3 prolapse, mid AMVL prolapse	5
Patient 16	0	0	0	0	1	1	Surgery: P2 prolapse, mid AMVL prolapse	5
Patient 25	0	0	0	1	2	1	TEE: P2 flail, P3 prolapse	6
Patient 27	0	0	0	0	2	1	TEE: P1/P2 flail, focal prolapse AMVL tip	5

0 – Normal segment, 1 – Prolapsing segment, 2 – Flail segment

### Inter-observer and intra-observer variability

Inter-observer agreement for classification of leaflet abnormality was 96% (132 of 138 leaflet slices) and 97% (66 of 69 jets) for mitral regurgitant jet direction. Intra-observer agreement for classification of leaflet abnormality was 98% (136 of 138 leaflet slices) and 98% (67 of 69 jets) for mitral regurgitant jet direction. For inter-observer and intra-observer evaluation all 10 patients were correctly classified as having anterior, posterior or bi-leaflet prolapse and all 10 were correctly classified as suitable for mitral valve repair or replacement.

## Discussion

In this study of patients with mitral valve prolapse and known moderate or severe mitral regurgitation, a simple CMR protocol allowed systematic anatomic mapping of leaflet abnormalities. There was excellent concordance between CMR and transthoracic echocardiography in the identification of jet direction and leaflet abnormality.

Mitral valve repair is the surgical procedure of choice in patients with significant mitral regurgitation secondary to mitral valve prolapse. Disease localised to the posterior mitral valve leaflet or focal involvement of the anterior mitral valve leaflet is most amenable to mitral valve repair, whereas patients with extensive involvement of the anterior leaflet or incomplete closure of the valve are more suitable for valve replacement [5]. The presence of a flail mitral valve leaflet identifies patients who are at a higher risk of sudden cardiac death and may warrant early surgery if the valve is repairable [9]. Although TTE images using harmonic imaging can usually identify leaflet abnormalities in mitral valve prolapse, many patients will have poor image quality due to, reduced ultrasound penetration through scar tissue, air filled lung or excess adipose tissue [4]. Because of variation in image quality and imaging widows systematic segmental mapping of the mitral valve leaflets is often not attempted using 2-dimensional TTE in clinical practice. TOE has traditionally been the investigation of choice, as the shorter distance between the probe and posterior cardiac structures results in better resolution of the left atrium and mitral valve. However, trans-oesophageal echocardiography does have known risks from conscious sedation and oesophageal intubation, and accurate acquisition and interpretation of images is highly dependent on operator experience. CMR mapping of the mitral valve using a simple protocol can reliably acquire long axis images through the valve, facilitating localisation of leaflet abnormalities and regurgitant jet direction.

When compared to modern TTE, the CMR mapping protocol accurately identified the abnormal leaflet in 98% of cases and correctly identified the predominant mitral regurgitant jet direction in all but 1 patient. The difference between the 2 techniques was differentiating leaflet flail from prolapse in 3 patients and CMR failing to detect a borderline prolapse (2 mm) involving an anterior mitral valve leaflet.

When compared to findings at surgery or TOE, CMR mapping according to a 6 segment model, correctly classified the presence/absence of an abnormality in 49 out of 60 segments in 10 patients. The non-concordance was due predominantly to disagreements regarding presence or absence of mild degrees of prolapse. In one patient, TOE and CMR disagreed regarding whether the middle (P2) or medial (P3) segment was flail, and in another patient, a flail was seen on CMR, but was classified as a prolapsing segment on TOE. Discrepancies in segment classification of prolapse are likely due to slight differences in imaging planes used by TOE and CMR mapping. Using either technique, variation in defining the border between adjacent leaflet segments (e.g. A1 from A2) can lead to minor differences in classification but is less likely to effect the decision for valve repair versus replacement. The discrepancies in classification of prolapse and flail segments may also in part be due to superior spatial resolution of echocardiographic over CMR when there are adequate echocardiographic windows. CMR spatial resolution is dependent on the voxel size and the slice thickness of the planes used. In this study, the typical voxel size was 1.5 × 1.5 mm and a slice thickness of 6 mm. Hence, visualising the direction of the mitral valve leaflet tip (1 – 5 mm thickness dependent on the degree of mitral leaflet thickening) to define segment prolapse versus flail may be difficult. In addition, insufficient contrast between the signal loss defining the origin of the regurgitant jet and the distal mitral leaflet tip may contribute to the minor differences seen.

An advantage of CMR compared with TTE is that because there is no limitation of imaging windows the CMR mapping protocol enabled a complete and systematic assessment of the mitral valve in every patient. Contiguous slices perpendicular to the valve closure line were consistently and easily obtained producing a standardised data set for interpretation. The very high inter- and intra-observer agreement obtained reflect this. Acquisition of the mapping images was efficient, requiring on average 7 cine images, and between 5 to 10 minutes per patient.

In patients with mitral valve prolapse, CMR has an established role in the assessment of LV size and function and mitral regurgitation severity. With the addition of mitral valve mapping, CMR can potentially provide a comprehensive assessment of mitral regurgitation secondary to mitral valve prolapse in patients undergoing evaluation for valve surgery.

### Study limitations

A limitation of this study is that a gold standard mapping assessment, either operative findings or TOE imaging, was available in only one third of patients. A larger study with surgical and/or TOE corroboration is required to assess the accuracy of CMR in segmental mapping of mitral valve leaflet pathology.

The study cohort comprised patients with known mitral valve prolapse with at least moderate mitral regurgitation on prior echocardiography. The ability of systematic CMR assessment of the mitral valve to detect mitral regurgitation jet lesions or leaflet abnormalities in patients with mild mitral regurgitation or other leaflet pathologies was not examined. The utility of CMR in the evaluation of mitral regurgitation in patients with atrial fibrillation was not assessed. These patients were excluded due to concern regarding reduced image quality due to the irregular rhythm. In addition to mapping of the abnormal segments, mitral valve leaflet length, the presence of mitral annular calcification and the assessment of sub-valvular structures are often important in assessing the ability to repair the mitral valve. These parameters were not formally assessed in this study. Whilst CMR is likely to be useful for assessment of mitral leaflet length and potentially the three-dimensional relationships between leaflets and subvalvular structures, the assessment of calcification by CMR is poor.

## Conclusion

In patients with mitral regurgitation due to mitral valve prolapse, assessment of the mechanism of mitral regurgitation, jet direction and systematic valve mapping using a simple and efficient CMR protocol is feasible and should be considered as part of the standard CMR examination in patients with mitral regurgitation. Further studies are required to evaluate whether CMR valve mapping could be an alternative when preoperative TOE mapping is required.

## Abbreviations

**TTE **– trans-thoracic echocardiogram, **TOE **– trans-oesophageal echocardiogram, **CMR **– Cardiovascular Magnetic Resonance, **AMVL **– Anterior Mitral Valve Leaflet, **PMVL **– Posterior Mitral Valve Leaflet

## Competing interests

The author(s) declare that they have no competing interests.
